# A case report of a rare form of calcium pyrophosphate disease: sacroiliitis with soft tissue involvement mimicking an infectious disease

**DOI:** 10.1093/rap/rkae123

**Published:** 2024-10-04

**Authors:** Alexia Leloix, Marion Hervouet, Émilie Chotard, Raphael Guillin, Pascal Guggenbuhl, François Robin

**Affiliations:** Rheumatology Department, Rennes University Hospital, Rennes, France; Rheumatology Department, Rennes University Hospital, Rennes, France; Rheumatology Department, Rennes University Hospital, Rennes, France; Department of Medical Imaging, Rennes University Hospital, Rennes, France; Rheumatology Department, Rennes University Hospital, Rennes, France; Rheumatology Department, Rennes University Hospital, Rennes, France

Key message

- Crystal-induced arthritis disease can mimic infectious arthritis in the sacroiliac joint with soft tissue pseudo-abscesses.

Dear Editor, We report a 75-year-old man with a known history of calcium pyrophosphate disease for several years located on wrist, elbow and shoulder bilaterally. He was admitted in the rheumatology department of Rennes University Hospital for acute right hip pain. This pain appeared suddenly, a few days before admission to hospital, associated with fever and functional disability. At the same time, the patient also had functional irritative urinary complaints with dysuria and urethral discharge. The patient walked with an antalgic gait. On palpation of the S1 spinous process, right buttock pain that radiated to the thigh was reproduced. However, the range of hip and spinal mobility was normal.

Blood tests at admission showed biological inflammation (CRP 251 mg/l, white blood cell count 16.7 × 10^9^/l with 14.5 × 10^9^/l neutrophils). Blood culture and urine samples were sterile. Abdominal and pelvic CT scans showed prostatitis, which led to the prescription of ceftriaxone.

Despite a few days of effective antibiotic therapy, the patient remained markedly limited in walking, with persistent pain in the right lower leg. Careful reassessment of the CT scan revealed some macro-abscesses in the soft tissues around the SI joint with an associated aspect of right sacroiliitis (Supplementary Fig. S1, available at *Rheumatology Advances in Practice* online). Ceftriaxone was discontinued in order to perform joint and abscess aspirations. This joint aspiration was made 7 days after the antibiotic therapy was discontinued. Synovial fluid culture was sterile, as well as 16S rRNA gene sequencing. Microcrystal analysis revealed plenty of calcium pyrophosphate crystals.

Antibiotics were therefore not reintroduced. Another pelvic CT scan and MRI was performed 3 weeks later with persistent significant abscesses in the soft tissue, with stability of the bone lesion of the right SI joint (Fig. 1 and Supplementary Fig. S1, available at *Rheumatology Advances in Practice* online). MRI showed an extension to the contralateral side with onset of liquid in a giant bone geode. A bone biopsy was performed 4 weeks after the first CT. Direct examination and culture did not show any evidence of infection or malignancy. In parallel, the clinical course of the patient improved with resumption of walking, no more limp and no fever, without introduction of any treatment excluding analgesics.

**Figure 1.**
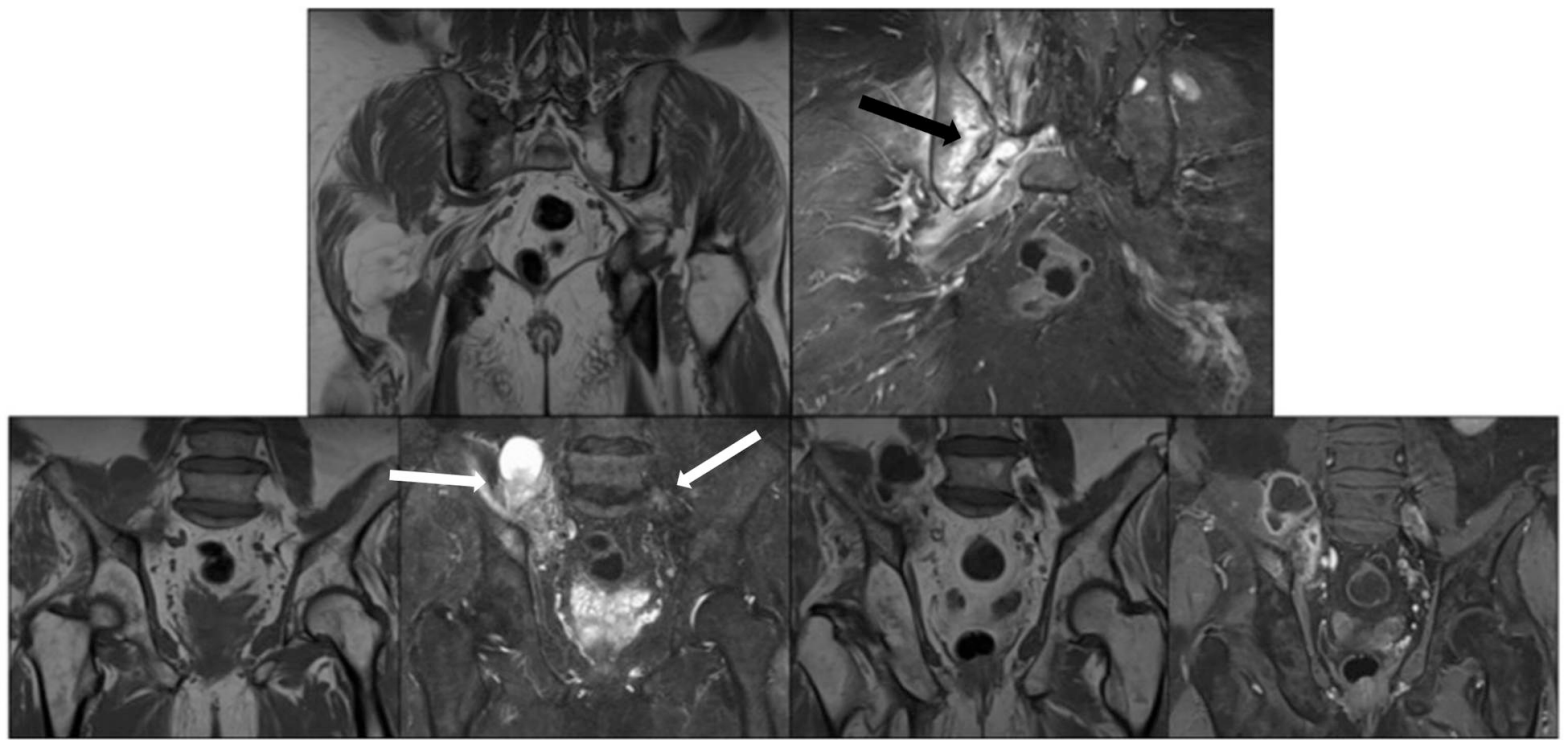
SI MRI evaluation during hospitalization with significant soft tissue extension. Upper line: T1-weighted sequence (on the left) and T2-weighted sequence (on the right) in the coronal section focused on the SI joints. Large sacroiliitis with extensive bone oedema (black arrow). Lower line from left to right: T1-weighted sequence, T2-weighted sequence, T1-weighted water sequence without gadolinium injection and T1-weighted water sequence with gadolinium injection showing abscesses in adjacent soft tissues (white arrows)

At the 3-month follow-up visit, there was no sign of clinic relapse, with complete resolution of symptoms. One year later, MRI evaluation showed complete resolution of abscesses and bone oedema, with stability of the bone macro-erosion. Investigation for a secondary cause of calcium pyrophosphate crystal deposition (CPPD) disease was negative.

To our knowledge, this is a rare example of sacroiliitis associated with macro-abscesses in soft tissues due to CPPD disease. Sacroiliitis diagnosis is based on clinical and radiological arguments. The two most common descriptions of sacroiliitis are spondylarthritis and infectious disease, particularly when it is unilateral. As a fibrocartilage, the SI joint is a potential site of CPPD [1]. CPPD disease more frequently affects the knees, wrists and pubic symphysis. SI involvement in CPPD is classical in textbooks but remains rare [2].

The imaging presentation is classically represented by a thin intra-articular calcium band [3]. Previous X-rays could have helped for diagnosis. But, in our case, the patient had not had any before. In our patient, the main imaging features were the presence of soft tissue pseudo-abscesses, highlighted in the CT and MRI assessments. However, abscesses in soft tissues are mainly described in infection or neoplastic causes. Pseudo-abscesses have been rarely described in CPPD disease [4, 5], as an aseptic muscular pyomyositis, possibly located in the psoas and the left obturator externus. An alternative clinical presentation as iliopsoas bursitis [6] can also be considered.

In our patient, infectious disease was first suspected. However, because of several and repetitive negative infectious samples, including a joint fluid culture, crystal-induced arthritis had to be considered [7]. In our patient, joint aspiration under CT scan revealed the crystals of CPPD.

This case describes a potential differential diagnosis of septic sacroiliitis and strengthens the need to wait for aspiration or bone biopsy results before starting antibiotic treatment in the absence of severe sepsis. In these specific cases, the contribution of dual-energy CT could potentially support the diagnosis of microcrystalline deposits [8].

In conclusion, crystal-induced arthritis disease can mimic infectious arthritis in the SI joint with soft tissue pseudo-abscesses. This observation highlights an atypical and rare form of CPPD disease and reinforces the need for a systematic search for crystals in all joint aspirations.

## Supplementary Material

rkae123_Supplementary_Data

## Data Availability

All data generated or analysed during this study are included in this article.
