# Supplementary material for: A case report of a rare form of calcium pyrophosphate disease: sacroiliitis with soft tissue involvement mimicking an infectious disease
Source: Rheumatol Adv Pract. 2024 Oct 4;8(4):rkae123. doi: 10.1093/rap/rkae123 (PMC11520397; doi:10.1093/rap/rkae123)
Supplement: rkae123_Supplementary_Data [file rkae123_supplementary_data.docx]

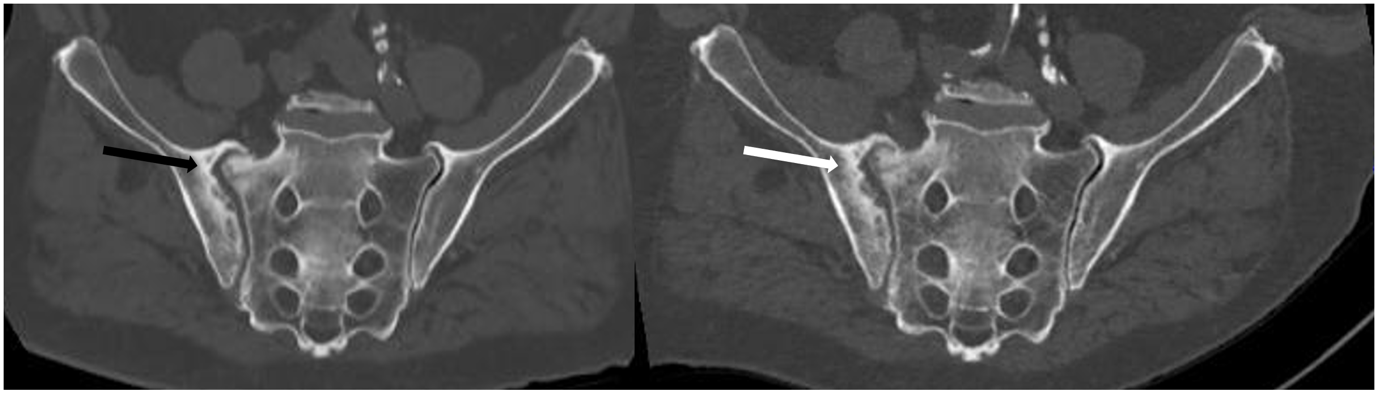


**Supplementary Figure S1: Sacro-iliac CT evaluation during hospitalization.** On the left, first computerized tomography (CT) in the axial section of the sacroiliac joint showing the right sacroiliitis (Black arrow). On the right, the second CT realized 3 weeks after admission, with significant evolution and increase of joint effusion (White arrow).
